# Pulsed-field ablation: Computational modeling of electric fields for lesion depth analysis

**DOI:** 10.1016/j.hroo.2022.05.009

**Published:** 2022-05-25

**Authors:** Daniel Meckes, Mehrdad Emami, Ian Fong, Dennis H. Lau, Prashanthan Sanders

**Affiliations:** ∗ Centre for Heart Rhythm Disorders, University of Adelaide and Royal Adelaide Hospital, Adelaide, Australia; † CathRx, Rydalmere, Australia

**Keywords:** Catheter ablation, Pulsed-field ablation, Irreversible electroporation, PFA doses, Tissue contact

## Abstract

**Background:**

Pulsed-field ablation (PFA) is an emerging and promising nonthermal technology for cardiac ablation. The effective applied voltage to achieve adequate irreversible myocardial injury is not well studied. The pulsed-field strength remains independent of tissue contact; therefore, PFA is assumed to be an ablation technology, not mandating the need for tissue contact.

**Objective:**

Determine the effect of applied voltage and distance to surface on depth of myocardial injury using PFA.

**Methods:**

A computational model was developed and validated based on extracted data from in vivo studies to examine the effect of different applied voltages and the impact of distance between the catheter and endocardial surface on the depth of irreversible myocardial injury using PFA.

**Results:**

The depth of lesions created by PFA are dose-dependent, and there is a direct correlation between applied PFA voltages and depth of irreversible myocardial injury. The minimum applied voltage of PFA required to create a lesion deeper than 1 mm is 300 volts. The catheter-tissue contact plays a pivotal role in determining lesion depth. With optimal catheter contact in the absence of trabeculation, the minimal applied energy required to achieve a 3-mm-deep lesion is 700 volts. A minor increase in the catheter-tissue distance of 1–2 mm doubles the minimum required applied voltage, increasing it to 1500 volts.

**Conclusion:**

PFA is an important new technology that is proposed to be more efficacious and safer than currently used thermal ablation. Here we demonstrate the impact of dose dependence and the need for maintaining tissue contact during ablation.

## Graphical abstract

Graphical Abstract: The association between applied voltage and tissue contact on the depth of the lesion

**Figure:**
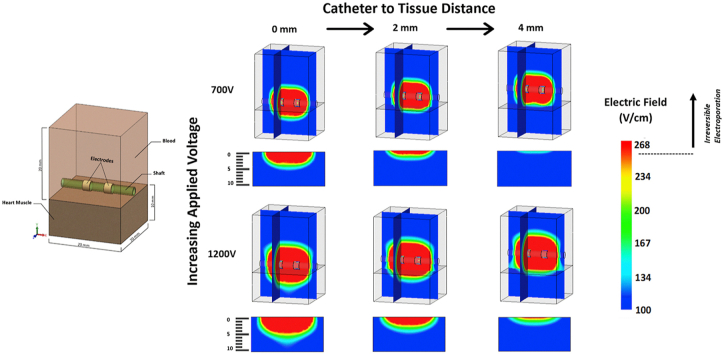

**Key Findings**

▪ Pulsed-field ablation (PFA) is an emerging nonthermal ablation technology. It is commonly believed that the catheter-tissue contact is nonessential in PFA, considering this technology’s nonthermal nature.
▪ Our computational model has shown a correlation between PFA doses and lesion depth, higher doses being associated with the deeper irreversible lesions.
▪ This modeling study demonstrated a reverse correlation between the lesion depth and the distance between the ablation catheter and endocardium. In some cases, by elevating the catheter 1–2 millimeters from the endocardium, the PFA dose required to achieve the same lesion depth was doubled.
▪ This computational modeling study paves the road for further in vivo studies investigating the safety and efficacy of different PFA doses.
▪ Despite the common belief, tissue contact is essential in PFA; however, performing in vivo studies is expected to be challenging owing to the complexity of cardiac anatomy.
▪ Further animal studies are required to investigate the effect of catheter contact in PFA.

## Introduction

The initial experimental animal studies of cardiac ablation by electroporation started in the early 1980s using intracardiac direct current shocks.^1^ The therapy disrupted the tissue's cell membrane, resulting in apoptosis and fibrosis.^2^^,^^3^ The generated pores in the cell membrane were documented using electron microscopy; hence, the technology was called electroporation.^4^ Today, electroporation by intracardiac direct current shock has evolved to a more precise therapy known as pulsed-field ablation (PFA). PFA is an emerging revolutionary technology that has been proposed to have several benefits over currently available thermal energy sources for cardiac ablation. Studies are suggestive of tissue selectivity with the creation of transmural and durable lesions while obviating the potential for collateral injury.^5^ Indeed, it is even proposed that the therapeutic delivery may not mandate direct tissue contact, given its use of electrical fields.^6^^,^^7^

Different groups have provided data on PFA use in preclinical and clinical settings.^6^^,^^8^ Although these reports have proprietary components, all studies have evidence supporting the above assertions. Therefore, there is considerable enthusiasm for the hope offered for safer and more effective techniques that are poised to transform the field. Nevertheless, the impact of several variables remains poorly studied.

In this computational modeling study, we use a model developed and validated against published variables of PFA to evaluate the effect of varying doses of PFA and determine the impact of tissue contact on lesion depth.

## Methods

### Development and validation of the model

**Figure 1:**
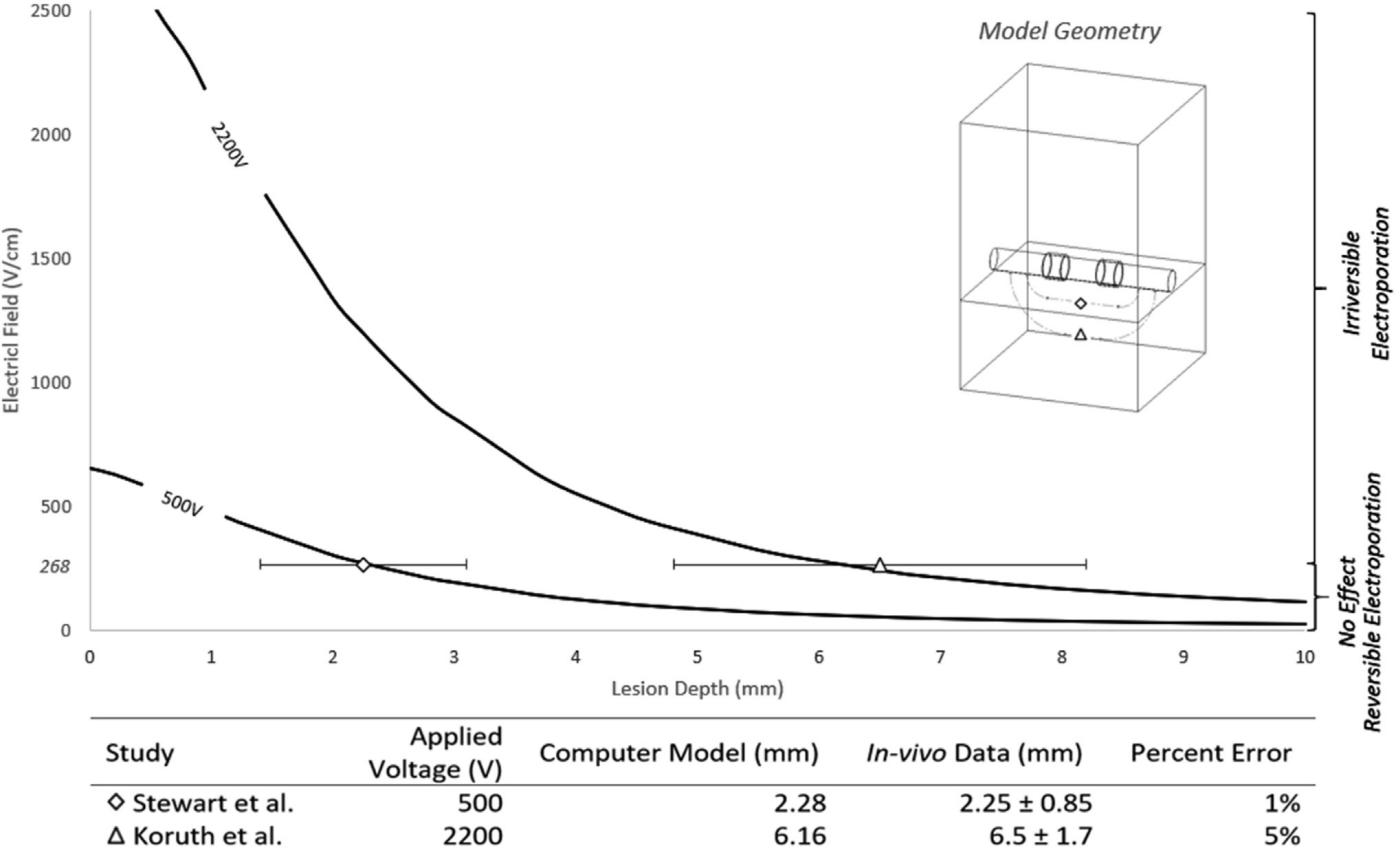
Computer model validation results. Validation results were obtained from available literature reporting lesion depth due to pulsed-field ablation delivery in porcine models. The graph assumes an irreversible electroporation threshold of 268 V/cm for the in vivo data. The computer model has a 1% and 5% error with Stewart and colleagues^6^ and Koruth and colleagues^18^, respectively.

We developed a computer model to study the effect of different PFA doses and evaluate the impact of tissue contact on the myocardial lesion depth. We based our model on a 2-electrode catheter configuration to simplify computation (see Graphical Abstract and Figure 1 – model geometry). To examine the principles of PFA, the 2-electrode model is adequate, and the findings of this model are generalizable to most catheter shapes and configurations. The computational domain includes a simplified 2-electrode catheter model placed in the blood pool adjacent to the endocardium to simulate the in vivo intracardiac environment. Figure 1 shows the geometry located in the upper right corner of the figure, with an expanded description provided in the Supplement.

We used a simplified model using 2-mm-long electrodes, having 4 mm interelectrode spacing, as previously described.^9^ This electrode size and orientation shows deep uniform electric fields in the myocardium. In this model, we examined the effect of different applied voltages on the depth of the lesion. Furthermore, we elevated the catheter from the endocardial surface to evaluate the impact of tissue contact on the lesion depth.

### Simulation of different applied voltages

To simulate the effect of different applied voltages on the depth of the myocardial lesion, the modeled electrodes were charged from 100 volts to 2500 volts in 200-volt increments, and the depth of lesion was studied for every given applied voltage.

### Simulation of electrode contact

The modeled 2-electrode catheter assembly was elevated above the endocardial surface at 1-mm intervals up to 5 mm to simulate the effect of catheter-tissue contact. In this case scenario, the geometry is intended to simulate no electrode contact with the endocardium.

The simplified 2-electrode model can be verified using the electric field created by 2-point charges. The governing equation for the 2-point charge scenario is identified below:

(1) E=2kqa(a2+y2)32

In this equation, E is the electrical field; *k* is the proportionality constant; *q* is the applied charge; *a* is ½ the distance between the point charges, and *y* is the distance along the y-axis, where the electric field is in units of newtons per coulombs (N/C) that is directly equivalent to the SI units of volts per meter (V/m), which can be further converted into volts per centimeter.

The 2-point charge calculation and the computer model show distinct similarities when evaluating the resulting electric field displacement and strength. When the 2-point charge equation is prescribed similar input geometries as the 2-electrode model, the electric field decreases, having an inverse-square drop-off like the computer model.

Unlike the 2-point charge equation (1) used for verification, Gauss’s law governs the computational domain, and the more mathematically complex Maxwell’s equations are used to compute the added tissue properties. To solve such complex numerical calculations, the ElectroMagneticWorks multi-core iterative electrostatics solver is used (EMWorks, Lachine, Canada). This finite element method software computes the electric (E) and vector displacement fields (D) including the divergent (∇×) and curl operators (∇⋅), charge density (ρ), material permittivity (ε), and electric potentials (φ) that form the famous Poisson equation:

(2) ∇⋅(ε∇φ)=ρ

As we demonstrate in this current report, the Poisson equation (2) can be used to solve for a given PFA catheter assembly and its input boundary conditions. The software imposes boundary conditions such as the amplitude of electric potential being constant on the electrode surface and the electric field vector being parallel to insulative surfaces such as the insulated catheter shaft.^10^ Without the aid of finite element method computer programs, analytically computing the above problem becomes quite challenging. Physical properties are specified below.

#### Material properties

We elected to use gold electrodes to resemble one of the available catheters in the market.^6^ We chose a nonconductive polymer for the shaft of the modeled catheter. The details of the material properties and input boundary conditions are provided in the Supplement.

Predicting tissue conductivities is challenging, as it may change owing to temperature and local electric field intensity.^10^ PFA is associated with a rapid temperature burst of a few degrees.^10^ This temperature rise is negligible with the right PFA waveform (voltage amplitude, pulse width, and the number of pulses delivered).^7^ However, as local tissue temperature gradients change owing to resistive heating (joule heating), the thermal and electrical conductivities of the tissue also change. Such changes to myocardial conductivity owing to temperature rise are accounted for in this model and are described in detail in the Supplement.

#### Irreversible electroporation

Electroporation has a graded effect, and the cell injury caused by electroporation ranges from reversible electroporation with temporary pore formation in the cell membrane to irreversible electroporation (IRE) associated with cell death by apoptosis. The cellular effect of electroporation is defined mainly by the resulting electric field, which depends on the applied voltage, the frequency of pulses, the pulse polarity (monophasic vs biphasic), and the catheter’s proximity to the tissue.^11^, ^12^, ^13^ The cellular effect of electroporation is also governed by tissue thickness, cell membrane action potential, and tissue homogeneity.^14^^,^^15^ The changes in myocardial conductivities were estimated using available data and accounted for in this model to simplify the complex association between electroporation and its tissue effect. For a healthy sheep myocardium, the field strength required for IRE was defined as 268 V/cm, as previously reported.^9^^,^^16^ The IRE threshold of 268 V/cm is derived from an animal model that used a unipolar pulse delivery. When delivering PFA using oscillating bipolar squared waveforms, the IRE threshold could be different.^17^ A detailed explanation for choosing the IRE threshold of 268 V/cm in this computational modeling is provided in the Supplement.

#### Pulse waveform

Electrical pulses can be delivered in various forms, including but not limited to *sine, square, triangle*, or *sawtooth*. A *sine* waveform produces a pulse amplitude that changes in a sinusoidal fashion. As for a *triangle* waveform, the pulse duration is shorter than the pulse rise and fall time, creating a sharp angle. With a *sawtooth* pulse, the pulse amplitude increases sharply and then declines gradually. Today, PFA is typically delivered using frequent short-duration square pulses. Although a symmetrical square wave is ideal, voltage rise and fall times do not occur instantaneously. Therefore, higher-frequency waveforms or faster pulse durations form a triangular shape—especially when high-voltage amplitudes are used. This is because the voltage must climb to higher amplitudes but at slower durations than the pulse plateau. All of the described pulses can be delivered in a unipolar or bipolar fashion, with bipolar biphasic the preferred option. Although we present voltage as the PFA dose metric, current or current density mapping may be used rather than voltage and electric field analysis. For the current computational modeling, a high-frequency bipolar biphasic squared waveform is captured throughout with a PFA ablation threshold discovered using a low-frequency monopolar monophasic (sawtooth) waveform (more details are provided in the Supplement).

### Model validation

An in vivo model validation using a 2-electrode catheter using different PFA field strengths was not in the scope of this study. Therefore, the computational model in this study is validated against currently available published data acknowledging all the limitations of differences in the catheter design and configuration and the differences in PFA pulse numbers and durations. We extracted PFA lesion depth data from 2 studies that evaluated different field strengths in the atria and ventricles and validated our models based on their findings.^6^^,^^18^

### Experimental protocol

After validation of the model, we undertook simulations to characterize the depth of IRE as follows: The 2-electrode model was charged using a range of applied voltages from 100 to 2500 volts to illustrate the effect of applied voltages on the lesion depth. The simulations modeled the electrical fields at varying tissue depth at each dose to establish the lesion of IRE. An upper dosage of 2500 volts was selected in keeping with the descriptions used in the literature.

The same simulations were then repeated at varying degrees of tissue contact ranging from 0 mm to 5 mm from the tissue-catheter interface.

### Statistical analysis

In this computational modeling, all the continuous variables that were extracted from the published animal data are presented as mean ± standard deviation. The confidence intervals were calculated based on the number of animals in each study.

## Results

### Validation of the computational modeling

The computational model was validated against the in vivo PFA lesion depth data from 2 studies that evaluated different field strengths in the atria and ventricles. Figure 1 illustrates the validation of our computer modeling. The y-axis represents the field strength, and the x-axis represents a unit increase in tissue depth. At the catheter-tissue interface, the field strength is at its greatest value. However, as tissue depth increases, the resultant electric field strength depreciates in an inverse-square fashion as determined by our verification 2-point charge model (Figure 1).

Stewart and colleagues^6^ achieved an atrial lesion depth of 2.25 ± 0.85 mm (95% confidence interval [CI], 1.35–3.14) using a prescribed 500 V application of PFA. The calculated lesion depth was based on an IRE threshold of 268 V/cm, and the voltage load of 500 V in our model was 2.28 mm. This result is in keeping with the above-mentioned animal study results and demonstrates a 1% margin of error compared to the in vivo data. When we used a different prescribed voltage of 2200 V that was used in the ventricles by Koruth and colleagues^18^, our model predicted IRE to the depth of 6.16 mm, which compared well with the reported clinical data, which showed a lesion depth of 6.5 ± 1.7 mm (95% CI, 3.8–9.2), equating to a 5% margin of error. Our computational model’s lesion depth prediction is very well correlated with the histopathological data extracted from animal studies with a small margin of error.

### Impact of the applied voltage dose on lesion depth

**Figure 2:**
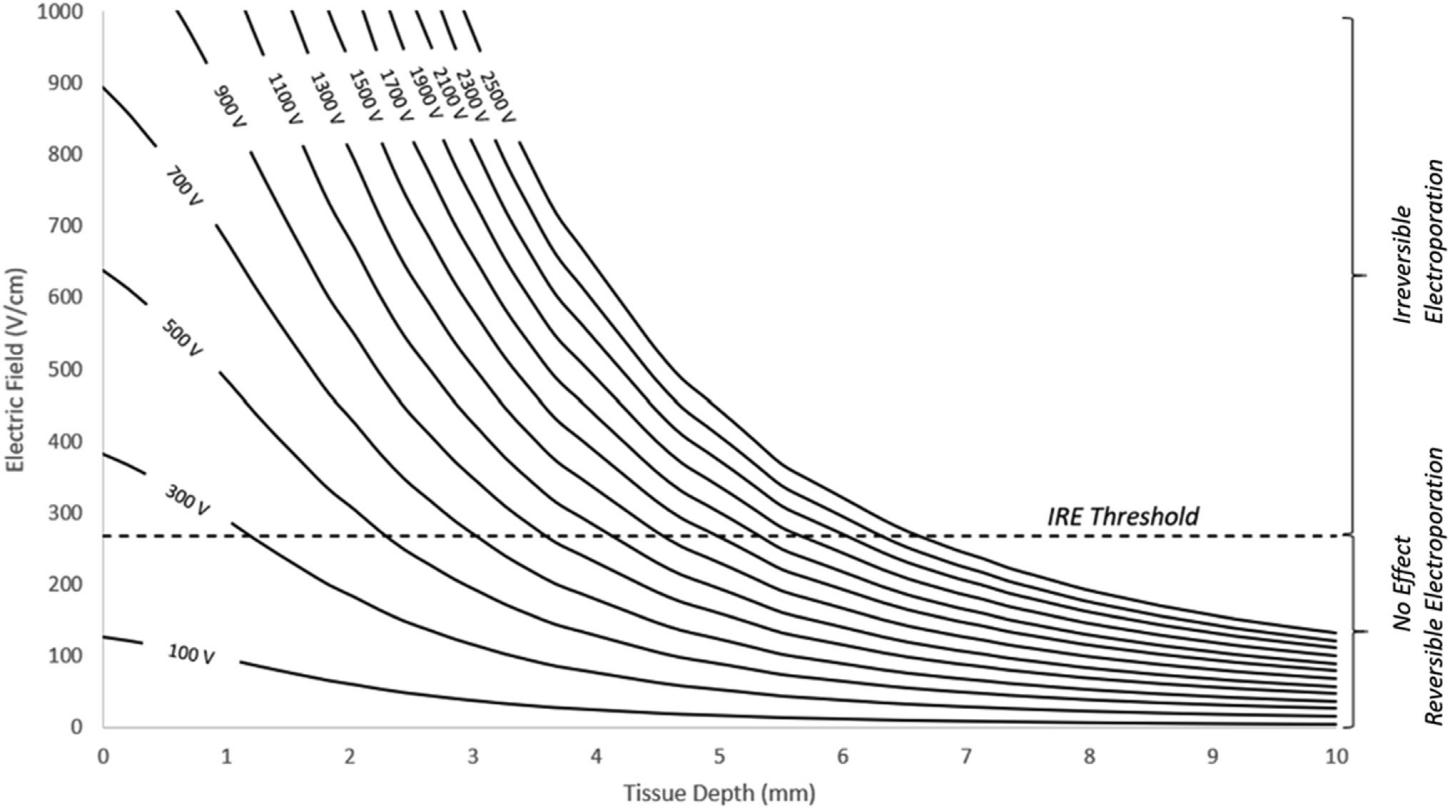
Electric field depths are evaluated under varying voltage loads. From 100 to 2500 V, the electric field strength (V/cm) is measured in the heart muscle, moving away from the electrodes. An irreversible electroporation (IRE) threshold is provided to estimate the boundary of irreversible and reversible electroporation.

The effect of the PFA dose on the tissue delivery of electroporation is shown in Figure 2. Using our model, we evaluated PFA doses of 100–2500 V with increments of 200 V. Based on this computational model, with good tissue contact, PFA voltages of less than 300 V would hardly have any irreversible effect on the myocardial tissue. As the voltage increases above 500 V to 2500 V, the effective lesion depth increases from 2.5 mm to 6.5 mm. It is very hard to achieve any effective irreversible myocardial injury deeper than 7 mm with PFA field strength of 2500 V and excellent contact with homogeneous tissue. Figure 2 expands on the data of the range of applied voltages evaluated and demonstrates the crossing when IRE is achieved to determine the expected lesion depth.

### Impact of the catheter-tissue contact on lesion depth

**Figure 3:**
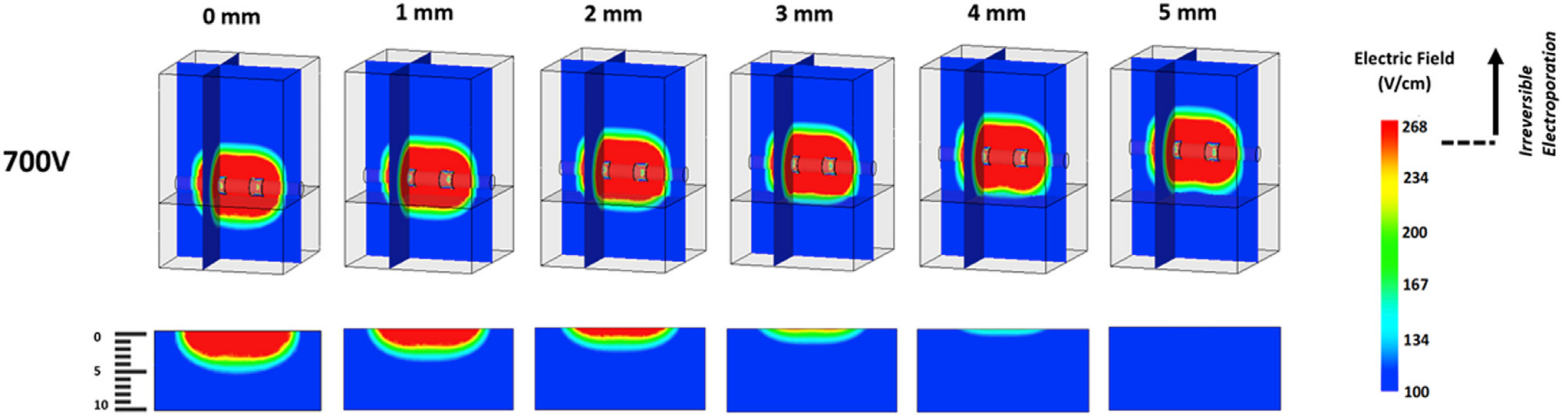
Electric field maps having a 700 volt load. The top complete models are presented in a dimetric orientation, having an electric field map plotted along the frontal plane running through the central axis of the electrodes as well as an electric field map plotted along a sagittal plane through the left electrode. The partial bottom models show the electric field map plotted on the frontal plane of the heart tissue alone. Electrodes are shown at adequate tissue contact and then raised above the tissue surface at 1-mm intervals from 1 to 5 mm from right to left.

**Figure 4:**
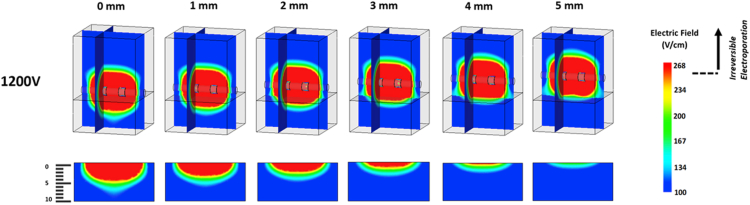
Electric field maps having a 1200 volt load. The top complete models are presented in a dimetric orientation, having an electric field map plotted along the frontal plane running through the central axis of the electrodes as well as an electric field map plotted along a sagittal plane through the left electrode. The partial bottom models show the electric field map plotted on the frontal plane of the heart tissue alone. Electrodes are shown at adequate tissue contact and then raised above the tissue surface at 1-mm intervals from 1 to 5 mm from right to left.

There was a reverse correlation between the catheter’s distance to the myocardium and the depth of the lesion. In this model, we charged the 2-electrode catheter with a fixed voltage (eg, 700 V and 1200 V) and examined the lesion depth for individually applied catheter-tissue contact scenarios. The catheter was elevated from the myocardial surface by 1-mm increments up to 5 mm. The lesion depth was evaluated for 2 variables, applied voltage and catheter-tissue contact. As shown in Figure 3, the effective irreversible myocardial lesion depth decreases with poor catheter-tissue contact. The top images in Figure 3 show the consistency of PFA field strength irrespective of tissue contact. This highlights the inherent differences between thermal ablation technology, in which the thermal energy decreases and dissipates in the blood pool in the absence of good contact, and PFA with a constant volume of energy irrespective of tissue contact. However, as illustrated in the partial model in Figure 3, the lesion depth using PFA significantly decreases by a 1-mm increment in the distance between the catheter and the myocardial surface. This principle remains valid for higher voltages, as illustrated in Figure 4. In this model, the applied voltage increased to 1200 V; however, the depth of irreversible myocardial injury is negligible when the catheter and myocardial tissue distance is 4 mm or more.

### Lesion depth: Combined impact of dose and contact

**Figure 5:**
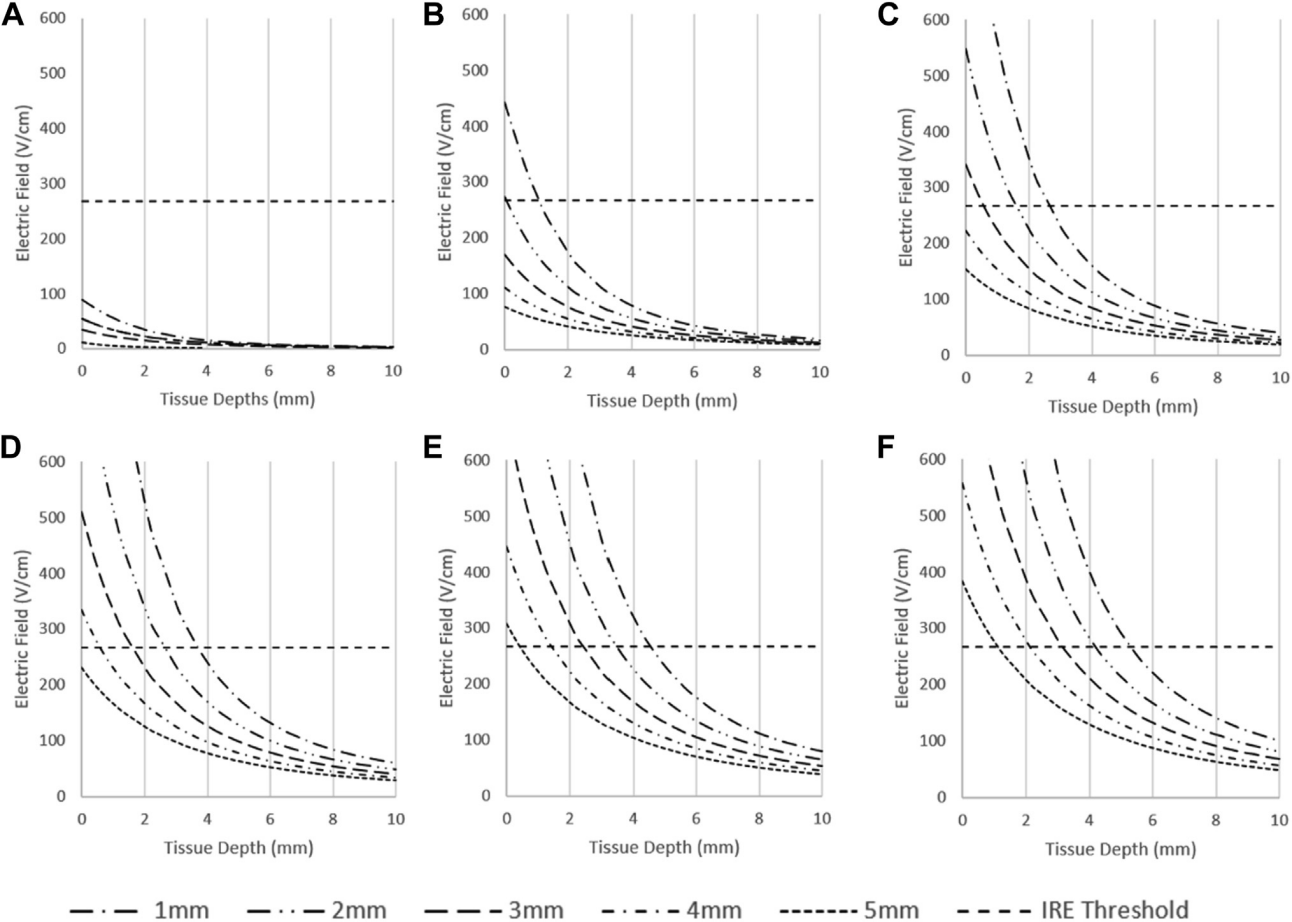
Electric field depth due to poor electrode-tissue contact. Electrodes are elevated above the tissue at 1-mm intervals from 1 to 5 mm above the tissue. The following voltages are evaluated under the above conditions: **A:** 100 V, **B:** 500 V, **C:** 1000 V, **D:** 1500 V, **E:** 2000 V, and **F:** 2500 V. An irreversible electroporation (IRE) threshold is given to estimate potential pulsed-field ablation lesion depths.

After evaluating the impact of tissue contact and PFA doses separately, we modeled the PFA dose and tissue contact in a single model. As shown in Figure 5, this model examines the correlation between tissue contact and PFA dose. In this model, we incorporated 6 doses of PFA: 100 V, 500 V, 1000 V, 1500 V, 2000 V, and 2500 V. We examined the lesion depth with a catheter distancing from the endocardial surface from 1 to 5mm with 1-mm intervals. As Figure 5A illustrates, PFA of 100 V does not reach the IRE threshold at any distance from the myocardium. Figure 5B shows that PFA of 500 V would only achieve 1 mm lesion depth at a catheter closer than 1 mm to the myocardium. PFA of 1000 V would produce adequate lesion depth at 4 mm only if in good contact; therefore, this dose would be enough for thin parts of the atrial myocardium. However, a less than 3 mm lesion depth is achieved only at a poor tissue contact of 1 mm. Similarly, the 1500 V PFA delivery will create a lesion to the depth of about 3 mm if the catheter is placed in less than 2 mm proximity to the myocardial surface (Figure 5D). PFA doses between 2000 V and 2500 V within 2 mm proximity achieve irreversible myocardial apoptosis with a lesion depth of about 3.5–4mm (Figure 5E and 5F).

## Discussion

### Major findings

PFA promises to potentially revolutionize catheter ablation with suggestions of improved safety and efficacy. We use computational modeling to evaluate the characteristics of the predicted lesions that result from PFA. We identify the following features of PFA: (1) There is a dose-dependent impact on the depth of tissue lesion. Using the critical zone of irreversible myocardial tissue damage, we can predict the lesion depth achieved by various doses of PFA. (2) Tissue contact or distance from the surface will continue to be an important feature of techniques that use PFA. Lesions due to PFA are field-dependent, with a significant drop-off when energy is delivered further away from the tissue surface.

These findings highlight that although PFA represents a new paradigm of ablation, tissue contact and dosage will remain important features in achieving transmural lesions. These findings have important implications for the use of PFA as an energy source and the appropriate design of delivery systems to ensure adequate tissue contact.

### PFA dose and lesion size

Several human and animal studies have shown that higher doses of PFA achieve an increased rate of pulmonary vein isolation (PVI) compared to low and intermediate doses.^19^ The first use of PFA in humans showed that the doses required for the acute left atrial (LA) posterior wall isolation were much higher than the doses needed for the acute PVI. The investigators of this study achieved 100% acute PVI using PFA 900–1000 V in the antrum of the PVs. The PFA dose required to achieve LA posterior wall isolation was 2100–2400 V.^5^ The electrical isolation of PVs and the posterior wall of the LA were used as a surrogate marker of lesion transmurality. The difference in PFA voltage required to isolate PVs vs LA posterior wall could be due to the difference in the wall thickness. The findings of this study are in keeping with our results showing the energy required to isolate thicker parts of the LA, namely the ridge between the posterior aspect of the LA appendage and anterior aspect of the left superior PV, and mitral isthmus was higher than the antrum of the PVs. When the same doses of PFA were trialed in a chronic model, the success rate of PVI at 3 months was not very high using the lower dose (900 V) of PFA^19^; however, a durable PVI was achieved using higher doses of PFA (1800–2000 V). This suggests that acute PVI at lower PFA doses was not due to irreversible myocardial injury, which required higher dosing as modeled in our study. Koruth and colleagues^20^ have studied low PFA doses vs high PFA doses for PVI in a swine model showing a higher success rate of durable PVI and deeper lesions with high doses of PFA.^20^ To the best of our knowledge, all the published data to date are consistent with our findings and demonstrate a dose dependency of the success rate of myocardial ablation using PFA.

### Tissue contact and lesion size

The successful myocardial ablation using thermal energies is dependent mostly on adequate catheter-tissue contact. Thermal energy could dissipate in the blood pool if the catheter is not in good contact with the myocardial surface, reducing the efficacy of thermal ablation. However, PFA acts on volume and does not require direct tissue contact.^7^ Confirmation of adequate and stable tissue contact can be challenging in closed-chest ablations. When PFA was used for the right atrial appendage (RAA) ablation, achieving good and stable tissue contact for all the electrodes of a circular ablation catheter was an impossible task considering the trabecular structure of RAA. Nevertheless, Stewart and colleagues^6^ achieved transmural lesions in RAA, concluding that tissue contact is not critical when PFA is used. Stewart and colleagues’ findings are explained with our model showing a thin-walled RAA with a less than 2 mm separation between the PFA delivery catheter and endocardium; achieving a full-thickness lesion is possible if adequate PFA voltage is applied. In another attempt to ablate ventricular myocardium using PFA,^18^ 91% of the lesions in the right ventricles were transmural, while none of the left ventricle (LV) lesions were transmural. The lack of transmurality in the LV could be related to the higher wall thickness of the LV, inadequate tissue contact owing to the trabecular structure of the LV, and higher LV contractility leading to poor catheter-tissue contact. Our computational modeling confirms the ventricular ablation findings using PFA, showing that when the catheter is displaced further than 2 mm from endocardium, the lesion depth created by PFA reduces significantly.

### Clinical implications

Catheter ablation is poised to undergo a paradigm shift with the advent of PFA. While demonstrated to result in instantaneous lesions with unparalleled boundaries in collateral injury, this computational model study demonstrates that the optimal dose of energy used needs to be determined in clinical use. In addition, clinicians will need to continue maintaining tissue contact during energy delivery to optimize lesion formation. In addition, it has important implications for the platforms used for delivery of PFA, as care will need to be afforded to ensure tissue contact during delivery.

### Limitations

This study represents the best modeling information that is available for evaluating PFA. Although it is based on the published literature and has been validated against reported observations, there remain a few key limitations. Firstly, the IRE threshold for cardiomyocytes to illustrate the PFA lesion is not well defined in the literature. Despite this limitation, we present the first validated computational model using typical PFA frequencies. We acknowledge the inherent limitation of validating a computational model against published data, including differences in catheter design and configuration and the differences in PFA pulse duration and pulse numbers. Another limitation lies within the validation metrics. The work proposed by Stewart and colleagues shows that 76% of lesions were transmural, meaning the actual lesion depth was not adequately captured by this study. Our model predicts this scenario showing a deeper lesion than Stewart’s work yet a shallower lesion than shown by Koruth and colleagues, where a maximum lesion depth was achieved. Considering this limitation, the model may offer a conservative, yet accurate, predictor to the actual lesion depth. However, these findings need further confirmation in in vivo and clinical studies.

## Conclusion

PFA is an important new technology that is proposed to be more efficacious and safer than currently used thermal ablation. Here we demonstrate the importance of determining the optimal dose of energy for ablation and the need for maintaining tissue contact during energy delivery to achieve durable and transmural lesions.
