# Supplementary material for: Pulsed-field ablation: Computational modeling of electric fields for lesion depth analysis
Source: Heart Rhythm O2. 2022 May 25;3(4):433–40. doi: 10.1016/j.hroo.2022.05.009 (PMC9463712; doi:10.1016/j.hroo.2022.05.009)
Supplement: Supplementary Data [file mmc1.docx]

**SUPPLEMENT**

**Material Properties**

The material properties used in the present model are listed in Table 1. Gold electrodes were used to reflect Medtronic's PVAC Gold™ catheter (Medtronic, MN, USA) currently being used for PFA delivery (1). Other materials such as platinum-iridium were considered, but no noticeable differences in the electric field profiles were observed. Although any non-conductive polymer could function as the catheter shaft, polytetrafluoroethylene (PTFE) was randomly selected from the EMS database and used throughout the study. In this computer model, we were particularly interested in insulating the shaft from the PFA electrodes. Figure 1 shows the labeled tissue properties and model geometry.

**Figure 1 (Supplement)**


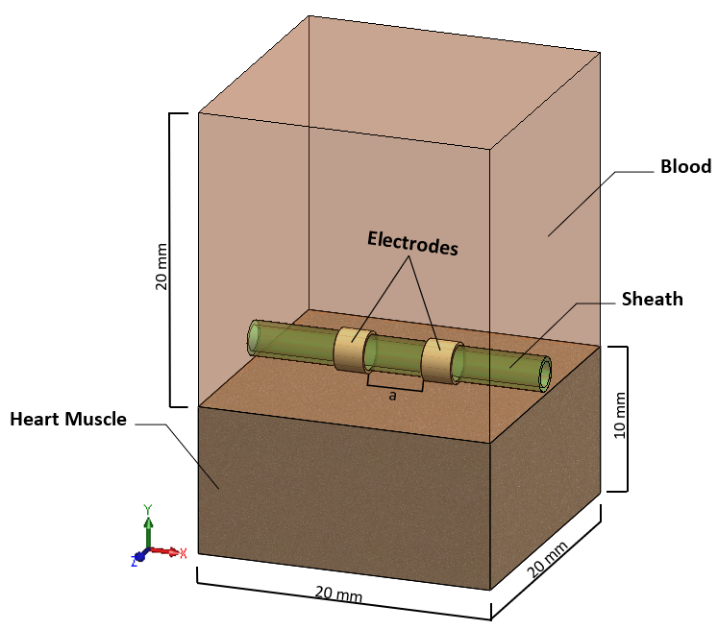


Tissue conductivities are challenging to predict as they are a function of local electric field intensity and temperature (2). For the myocardium, the temperature-dependent piecewise thermal conductivity function grows linearly 0.12°C-1 up to 100°C and is kept constant (3-5). Alternatively, heart tissue's electrical conductivity features an exponential growth of 1.5°C^-1^ between 0 and 100°C (3, 5, 6). Although temperatures beyond 100°C are not likely in PFA, heart tissue does experience a linear decay of 4 orders of magnitude for 5°C that models the tissue desiccation at 100°C, and then remains constant (3, 5, 7). To compound the problem further, experimental values for tissue conductivities may also increase due to continued pore formation where the cytoplasm opens previously unavailable intracellular current pathways (8, 9). This phenomenon likely relates to the waveform type, including monopolar or bipolar pulse deliveries and the number of pulses. For example, Tekle et al. reported that bipolar square waves permeabilized cell membranes better than unipolar square waves, and Garcia et al. illustrated the importance of higher pulse numbers leading to larger cell kill counts (10, 11). Such tissue conductivity and waveform effects can be analyzed experimentally and defined empirically, which can be combined with heating effects to form constituents of dynamic electrical conductivities (2).

To execute the heart muscle's dynamic behavior during PFA requires complex thermal-fluid modeling or difficult experimental measurements, then extensive ex-vivo tissue characterization data (2). Also, the overall goal of this work is to inform a PFA system design and provide insight into PFA lesion effects, potentially limiting the need for costly in-vivo testing. As such, this current report uses readily available ex-vivo electroporation data obtained from the kidneys (2).

Although the heart and the kidneys are quite distinct by way of function, looking deeper reveals tissue properties quite similar by comparison. Take, for example, tissue thermal conductivity, the heart muscle, and kidney tissue have thermal conductivities of 0.56 and 0.53 Wm^-1^°C^-1^, respectively (IT'IS database, Zurich, SWITZERLAND) (12). Therefore, we believe the electrical conductivity percent increases, as a function of temperature obtained in the kidney during ex-vivo electroporation, would provide a conservative yet relatively accurate alternative to heart muscle dynamic conductivities during PFA. In this respect, we chose a tissue conductivity at 45 kHz and approximated an 18% increase in conductivity, as might be expected when delivering >20 pulses. (12-14). These tissue properties would result from a waveform comprising two 15µs biphasic pulses and two 5 µs inter-pulse delays with added (0.0347 Sm^-1^) conductivity. This translates to about 150 kHz or very fast 3.325 µs biphasic pulses with no inter-pulse delays. As for the tissue permittivity, this was estimated based on typical values at the predetermined tissue conductivities. Similarly, the blood properties reflect the material properties occurring during the faster direct current (DC) PFA waveforms of 5µs pulse durations. However, the blood was neglected during electric field assessment as the electric fields in the blood are of little relevance to lesion assessment.

Supplementary Table 1 outlines the material properties used in the computer model:

**Table 1: Computer model material properties**

| Material | $\varepsilon$ (Fm^-1^) | $\sigma$ (Sm^-1^) |
| --- | --- | --- |
| Blood | 5120 | 0.703 |
| Heart Muscle | 7330 | 0.228 |
| Pure Gold | - | 41,000,000 |
| PTFE | - | 0 |

***Boundary Conditions***

Boundary conditions also reflect Neal et al. work where the anode is energized with a prescribed voltage, and the cathode is set to ground (2). In this current work, one electrode is energized while the other electrode is set to the ground. This boundary condition creates an electric field as the flow of current passes from one electrode and travels through the surrounding anatomical space and enters the grounded electrode. This study is a steady-state calculation, meaning pulse width and the number of pulses is not possible with this current analysis. Instead, the increased cell kill count due to waveform type is captured in the 18.5% increase to the material property's electrical conductivity and the resulting permittivity (2). As a result, the electrical field simulated between the two electrodes depicts the lesion depth due to the assumed heart tissue's dynamic conductivity taken from ex-vivo experimentation with kidneys (2). Further, this is made possible by a known IRE threshold of heart tissue of 268 V/cm (15). This also means that blood flow is not calculated for. Electric field profiles will not be affected by the lack of blood flow, particularly useful in thermal fluid modeling where passive cooling of electrodes is apparent.

***Irreversible Electroporation Threshold***

In this study, cardiac PFA lesions were identified using an IRE threshold of 268 V/cm. This threshold was first captured during unipolar monophasic PFA, which is indeed a markedly different waveform than is being employed by the clinical Medtronic and Farapulse systems used for validation purposes (15). Alternatively, a threshold of 400 V/cm is generally proposed (16-18). The selection of 400 V/cm as a threshold in previously published studies is based on in-vitro experimentation using rat myoblasts and defines an approximate threshold of 375 V/cm (19). The IRE threshold of rat myoblasts determined by Kaminska et al. is an incomplete data set suggesting an IRE threshold greater than 300 V/cm and less than or equal to 375 V/cm. Therefore, the current evidence indicates an average IRE threshold of myocardiocytes to be 322 ± 54 V/cm rather than 400 V/cm (15, 19).

Histopathology shows transitions from reversibly to irreversibly electroporated cells occur continuously throughout an electroporated lesion. Therefore, irreversible electroporation thresholds cannot be regarded as discrete values, nor can they be based on a single in vitro study(20). Instead, IRE threshold estimation is generally made by overlapping histological results with the computational model's electric field distribution (21, 22). Our computational model revealed an IRE threshold of 268 V/cm reported by Wittkampf et al. using a generalizable PFA waveform and tissue properties when overlapped with histological results reported by Medtronic and Farapulse (1, 23). In a recent study, Calouri et al. used their own PFA waveform combined with RFA tissue properties that are more conductive than those used in our computational model but reported the histological results remained superior to those predicted computationally (18). Although the authors contribute this to the effect of multiple pulses not accounted for in their model, it may also be due to the higher IRE threshold of 400 V/cm used to interpret the results as this would greatly reduce the IRE isotherm. A lower IRE threshold of 322 ± 54 V/cm would undoubtingly improve the computational prediction of the PFA boundary.

Finally, the waveform and pulse parameters used during in-vitro experimentation on rat myoblasts were also different from those currently employed by Medtronic and Farapulse. The waveform used was a monophasic square wave, whereas Medtronic and Farapulse currently use a biphasic square wave (1, 19, 24). Inasmuch, a biphasic square wave induces a more significant cellular response than a monophasic square wave (10). Additionally, a high-tilt exponential pulse, such as the one used by Wittkampf et al., may produce less injury than rectangular pulses (25). Therefore, there is reason to believe that cardiac myocytes may elicit an even lower IRE threshold than currently proposed by others (13, 8, 16, 24). For example, Oliveira et al. demonstrated that the rat ventricular myocyte experienced IRE at electric fields strengths as little as 50 V/cm (26). We suspect a typical IRE threshold induced by PFA to be greater than this value but less than 350 V/cm.

References:

1. Stewart MT, Haines DE, Verma A, Kirchhof N, Barka N, Grassl E, et al. Intracardiac pulsed field ablation: Proof of feasibility in a chronic porcine model. Heart Rhythm. 2019;16(5):754-64.

2. Neal RE, 2nd, Garcia PA, Robertson JL, Davalos RV. Experimental characterization and numerical modeling of tissue electrical conductivity during pulsed electric fields for irreversible electroporation treatment planning. IEEE Trans Biomed Eng. 2012;59(4):1076-85.

3. González-Suárez A, Berjano E, Guerra JM, Gerardo-Giorda L. Computational Modeling of Open-Irrigated Electrodes for Radiofrequency Cardiac Ablation Including Blood Motion-Saline Flow Interaction. PLoS One. 2016;11(3):e0150356.

4. Berjano EJ. Theoretical modeling for radiofrequency ablation: state-of-the-art and challenges for the future. Biomed Eng Online. 2006;5:24.

5. Avari H, Berkmortel C, Savory E. An Insight to the Role of Thermal Effects on the Onset of Atrioesophageal Fistula: A Computer Model of Open-Irrigated Radiofrequency Ablation. Cardiovasc Eng Technol. 2020;11(4):481-93.

6. Schutt D, Berjano EJ, Haemmerich D. Effect of electrode thermal conductivity in cardiac radiofrequency catheter ablation: a computational modeling study. Int J Hyperthermia. 2009;25(2):99-107.

7. Jain MK, Wolf PD. Temperature-controlled and constant-power radio-frequency ablation: what affects lesion growth? IEEE Trans Biomed Eng. 1999;46(12):1405-12.

8. Ivorra A, Villemejane J, Mir LM. Electrical modeling of the influence of medium conductivity on electroporation. Physical Chemistry Chemical Physics. 2010;12(34):10055-64.

9. Pavlin M, Kanduser M, Rebersek M, Pucihar G, Hart FX, Magjarevic R, et al. Effect of cell electroporation on the conductivity of a cell suspension. Biophys J. 2005;88(6):4378-90.

10. Tekle E, Astumian RD, Chock PB. Electroporation by using bipolar oscillating electric field: an improved method for DNA transfection of NIH 3T3 cells. Proc Natl Acad Sci U S A. 1991;88(10):4230-4.

11. Garcia PA, Davalos RV, Miklavcic D. A numerical investigation of the electric and thermal cell kill distributions in electroporation-based therapies in tissue. PLoS One. 2014;9(8):e103083.

12. Hasgall PA DGF, Baumgartner C, Neufeld E, Gosselin MC, Payne D, Klingenbock A, Kuster N,. IT'IS Database for thermal and electromagnetic parameters of biological tissue 2020 [updated july 30th, 2020. Available from: [www.itis.ethz.ch/database](file:///C:\Users\mehrd\Downloads\Writing%20in%20Progress\PFA%20Manuscripts\Computational%20Modelling-%20PFA-%201st%20Manuscripts\HRO2\www.itis.ethz.ch\database).

13. Stewart MT, inventorCardiac Pulsed Field Ablation patent 10,531,914. 2020 14 January 2020.

14. Viswanathan R, inventorSystems, apparatuses and methods for delivery of ablative energy to tissue. United States patent 15/334,646.

15. Wittkampf FHM, van Es R, Neven K. Electroporation and its Relevance for Cardiac Catheter Ablation. JACC: Clinical Electrophysiology. 2018;4(8):977-86.

16. Reddy VY, Koruth J, Jais P, Petru J, Timko F, Skalsky I, et al. Ablation of Atrial Fibrillation With Pulsed Electric Fields: An Ultra-Rapid, Tissue-Selective Modality for Cardiac Ablation. JACC: Clinical Electrophysiology. 2018;4(8):987-95.

17. Ramirez FD, Reddy VY, Viswanathan R, Hocini M, Jaïs P. Emerging Technologies for Pulmonary Vein Isolation. Circ Res. 2020;127(1):170-83.

18. Caluori G, Odehnalova E, Jadczyk T, Pesl M, Pavlova I, Valikova L, et al. AC Pulsed Field Ablation Is Feasible and Safe in Atrial and Ventricular Settings: A Proof-of-Concept Chronic Animal Study. Front Bioeng Biotechnol. 2020;8:552357.

19. Kaminska I, Kotulska M, Stecka A, Saczko J, Drag-Zalesinska M, Wysocka T, et al. Electroporation-induced changes in normal immature rat myoblasts (H9C2). Gen Physiol Biophys. 2012;31(1):19-25.

20. Handbook of electroporation. 1st ed. 2017. ed. Miklavčič D, editor. Cham, Switzerland: Springer; 2017.

21. Miklavcic D, Semrov D, Mekid H, Mir LM. A validated model of in vivo electric field distribution in tissues for electrochemotherapy and for DNA electrotransfer for gene therapy. Biochim Biophys Acta. 2000;1523(1):73-83.

22. Sel D, Cukjati D, Batiuskaite D, Slivnik T, Mir LM, Miklavcic D. Sequential finite element model of tissue electropermeabilization. IEEE Trans Biomed Eng. 2005;52(5):816-27.

23. Koruth JS, Kuroki K, Iwasawa J, Viswanathan R, Brose R, Buck ED, et al. Endocardial ventricular pulsed field ablation: a proof-of-concept preclinical evaluation. Europace. 2019.

24. Reddy VY, Neuzil P, Koruth JS, Petru J, Funosako M, Cochet H, et al. Pulsed Field Ablation for Pulmonary Vein Isolation in Atrial Fibrillation. Journal of the American College of Cardiology. 2019.

25. Tung L, O'Neill RJ. Comparison of electroporation thresholds of cardiac cell membranes by rectangular and exponential pulses. IEEE; 1995. p. 251-2 vol.1.

26. de Oliveira PX, Bassani RA, Bassani JW. Lethal effect of electric fields on isolated ventricular myocytes. IEEE Trans Biomed Eng. 2008;55(11):2635-42.
